# Ms. Claudete Moral retirement: our most profound appreciation for her dedication to ABO

**DOI:** 10.5935/0004-2749.2023-1002

**Published:** 2023-04-10

**Authors:** Eduardo M. Rocha

**Affiliations:** 1 Department of Ophthalmology, Otorhinolryngology, and Head and Neck Surgery Faculdade de Medicina de Ribeirão Preto, Universidade de São Paulo, Ribeirão Preto, SP, Brazil

**Figure 1:**
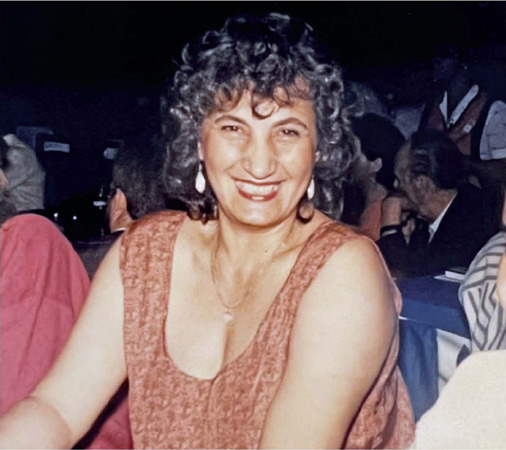
Claudete Moral at the Pan American Congress of Ophthalmology in Rio de Janeiro (1989).

Ms. Claudete Moral started working on the editorial process of *Arquivos Brasileiros de Oftalmologia* (*ABO*) in 1986. At that time, typewriters, stamps, and envelopes on her desktop were her work partners. Most of the actual readers and authors of *ABO* were not even born; she was dealing with papers and photos and connecting the authors and readers by exchanging physical messages via hundreds of paper sheets (Figure 1).

Over the course of 37 years, her office shifted several times. From Botucatu street (where ABO was housed in the *Escola Paulista de Medicina*) to Alameda Santos, the headquarters of the Brazilian Council of Ophthalmology (CBO), and later to Casa do Ator Street, the location of the new, modern office of *ABO*/CBO. Claudete always worked in Sao Paulo, Brazil, a big city where she chose to live, went to school, married, raised her daughter, and suffered and cheered with her beloved soccer team, Sport Club Corinthians Paulista.

Claudete faced more than just the challenges of modernization; during those decades, she served as administrative editor chair^(1)^. She worked with four editors-in-chief, thousands of authors and reviewers, and several publishing companies^(2)^.

Regular mail was replaced by facsimile (fax) and later by email to finally reach the modern online manuscript submission system. Claudete tested, checked, validated, and approved every single modernization step, following the best scientific journals in the world, and she guided new editors, authors, and reviewers throughout those changes during those four decades of editorial work. Around 20 years ago, Claudete shared the administration of *ABO* with her daughter, Claudia Moral, who learned the critical points of a reputed journal and knew how to manage the modernization as co-editorial manager.

Claudete, always respectful and generous with every author and potential author, represented the *ABO* in the Brazilian Meetings of Ophthalmology every year, in the business meeting with Scielo, with the CBO board of directors, and in the numerous editorial board meetings that took place during those years.

Now, Claudete goes home and takes some peace and quiet from the desperate messages and long phone calls requesting help on paper decisions. She may wish that all of our submissions were published, but she always worked with exemplariness and serenity.

We state here that our gratitude will never fully express our appreciation for her dedication that made the difference to *ABO*, the medical journal in which we are all proud to participate. Thank you, Claudete.

Eduardo M. Rocha

Editor-in-chief

ABO
